# The Association between the Australian Alcopops Tax and National Chlamydia Rates among Young People—an Interrupted Time Series Analysis

**DOI:** 10.3390/ijerph17041343

**Published:** 2020-02-19

**Authors:** William Gilmore, Tanya Chikritzhs, Hamish McManus, John Kaldor, Rebecca Guy

**Affiliations:** 1 National Drug Research Institute, Faculty of Health Sciences, Curtin University, GPO Box U1987, Perth, WA 6845, Australia; t.n.chikritzhs@curtin.edu.au; 2 Kirby Institute, University of New South Wales, Level 6, Wallace Wurth Building, High Street, Kensington NSW 2052, Australia; hmcmanus@kirby.unsw.edu.au (H.M.); jkaldor@kirby.unsw.edu.au (J.K.); rguy@kirby.unsw.edu.au (R.G.)

**Keywords:** alcohol policy, taxation, ready-to-drink beverages, alcopops, young people, chlamydia, interrupted time series analysis, autoregressive integrated moving average.

## Abstract

A national tax increase, which became known as the “alcopops tax”, was introduced in Australia on the 27th April 2008 on ready-to-drink alcoholic beverages, which are consumed predominantly by young people. The affordability of alcohol has been identified as the strongest environmental driver of alcohol consumption, and alcohol consumption is a well-known risk factor in the spread of sexually transmitted infections via its association with sexual risk-taking. We conducted a study to investigate whether there was any association between the introduction of the tax and changes in national chlamydia rates: (i) notification rates (diagnoses per 100,000 population; primary outcome and standard approach in alcohol taxation studies), and (ii) test positivity rates (diagnoses per 100 tests; secondary outcome) among 15–24 and 25–34-year-olds, using interrupted time series analysis. Gender- and age-specific chlamydia trends among those 35 and older were applied as internal control series and gender- and age-specific consumer price index-adjusted per capita income trends were controlled for as independent variables. We hypothesised that the expected negative association between the tax and chlamydia notification rates might be masked due to increasing chlamydia test counts over the observation period (2000 to 2016). We hypothesised that the association between the tax and chlamydia test positivity rates would occur as an immediate level decrease, as a result of a decrease in alcohol consumption, which, in turn, would lead to a decrease in risky sexual behaviour and, hence, chlamydia transmission. None of the gender and age-specific population-based rates indicated a significant immediate or lagged association with the tax. However, we found an immediate decrease in test positivity rates for 25–34-year-old males (27% reduction—equivalent to 11,891 cases prevented post-tax) that remained detectable up to a lag of six months and a decrease at a lag of six months for 15–24-year-old males (31% reduction—equivalent to 16,615 cases prevented) following the tax. For no other gender or age combination did the change in test positivity rates reach significance. This study adds to the evidence base supporting the use of alcohol taxation to reduce health-related harms experienced by young people and offers a novel method for calculating sexually transmitted infection rates for policy evaluation.

## 1. Introduction

Price changes, most commonly through taxation, have been studied more than any other governmental alcohol control policy with regard to their effect on consumption and related harms [1]. Through this research, the affordability of alcohol has been identified as the strongest environmental driver of alcohol consumption and related harms, particularly assaultive and road traffic injuries, both at the population-level and specifically among heavy drinkers and young people [1,2]. Natural experiments of alcohol price increases and decreases internationally, and systematic reviews and meta-analyses of these experiments, have confirmed this association [3,4].

Two national taxes introduced in Australia in the 2000s had significant but opposing effects on the price of ready-to-drink alcoholic beverages (RTDs), a category most commonly involving a pre-mixed and packaged combination of white or dark spirit and soft drink, which are consumed predominantly by young people [5,6]. In Australia, alcohol taxation falls entirely within the responsibility of the federal government; to our knowledge, there were no other substantial national tax changes or state/territory-level price changes (e.g., minimum unit price) for alcohol over the observation period [7]. First, under the goods and services tax (GST) introduced on the 1st July 2000, the tax rate on RTDs, previously the same rate as straight spirits, was reduced by 40% from $56.27 to $33.22 per litre of pure alcohol [8,9]. After the popularity and sales of RTDs increased in the intervening years and because of public concern over levels of alcohol consumption among young people, a 70% tax increase was introduced on the 27th April 2008 to bring the tax on RTDs back into line with straight spirits (from $39.36 to $66.67 per litre of pure alcohol) [10,11]. This targeted tax increase (commonly known as the “alcopops tax”) led some to argue that focusing on only one beverage type would merely lead to a substitution effect, whereby drinkers affected by the tax would simply switch to another beverage type, thereby rendering the tax ineffective as a public health intervention [9,12]. As it turned out, alcohol sales data from government and market research sources confirmed that both RTD sales and total alcohol sales began to decline immediately following the tax and continued to decline for at least two years. Increases in straight spirits sales were evident after the tax, but only fractionally offset the reduction in RTD sales [11,13,14]. Similar tax increases specific to RTDs were introduced across Europe in the 2000s, although few papers are published regarding their effects. In Germany, alcohol sales data indicated considerable subsequent reductions in RTD sales, with some evidence of partial substitution to straight spirits, but no overall reduction in total alcohol sales [15].

To date, all five studies that have evaluated the impact of RTD taxes on alcohol-related harms among young people have been conducted in Australia (across four states) and have examined associations with levels of emergency department (ED) attendance or hospitalisation [8,16,17,18,19]. Three studies conducted by a research group in the state of Queensland found no association between the alcopops tax and ED attendance or hospitalisation among 15–29-year-olds [17,18,19]. A New South Wales (NSW) study found that lowering the price of RTDs via the GST was associated with an increase in ED attendances among 18–24-year-old females. The subsequent increase in price due to the alcopops tax was associated with a decrease in ED attendances among 15–17, 18–24 and 25–49-year-old males and females, with the strongest association for 18–24-year-old females [8]. As the introduction of the alcopops tax coincided with the 2008 global financial crisis (GFC), which may have impacted disposable income, another important driver of alcohol consumption [20], the NSW study controlled for the effects of the GFC using monthly liquor retail turnover data [8]. A study of weekend nighttime ED injury attendances among males in Western Australia (WA) and Victoria also found significant associations with the alcopops tax. In WA, the new tax was associated with immediate decreases in injury among 12–19-year-olds and delayed decreases among 20–29-year-olds. In Victoria, immediate decreases in injury rates were seen among 15–19-year-olds with delayed decreases among 20–29-year-olds [16].

Alcohol use, particularly heavy drinking, is a well-known risk factor in sexually transmitted infection (STI) transmission via its association with sexual risk-taking, such as condomless sex and multiple casual partners [21,22]. Studies in the US and Canada have indicated subsequent reductions in STI rates following alcohol tax increases [23,24,25,26,27,28]. Evaluations of multiple tax increases on beer across the 50 US states in the 1980s and 1990s found decreases in gonorrhoea [25,26,27], syphilis [26] and AIDS [25] rates. An evaluation of the increase in real beer prices across the 10 Canadian provinces over the same period found decreases in chlamydia and gonorrhoea rates [28].

The most recent US studies examining the effects of a 2009 alcohol tax increase in Illinois and a 2011 alcohol tax increase in Maryland found reductions in chlamydia [24] and gonorrhoea [23,24] rates.

Despite using robust controlled interrupted time series [23,24,26,27] and interrupted time series designs [25,28], none of these studies controlled for the frequency of STI tests. The commonly accepted approach for estimating harm rates in studies of this nature is to rely on formal estimates of resident population. All studies of the association between alcohol price changes and STIs to date have relied on the resident population to estimate STI rates [23,24,25,26,27,28]. However, trends in chlamydia diagnoses are strongly correlated with the number of tests that occurred in that population during the same period and do not necessarily reflect the underlying prevalence in the population [29]. Testing behaviour itself can be influenced, for example, by sexual health promotion campaigns and is not equally spread across the population—considerable variation exists by age, gender and socio-economic status [30]. Therefore, when attempting to evaluate a policy, a more sensitive measure of trend in chlamydia rates might be achieved by applying counts of chlamydia tests rather than resident population numbers as a measure of exposure (i.e., denominator), similar to methods used in national STI surveillance reports [31] and sentinel surveillance systems [29,32].

From reports in the literature of significant government investment in chlamydia awareness and screening programs in Australia from 2005 [33,34] and large increases in both chlamydia tests per 100,000 population (112% among 15–34-year-olds from 2005 to 2010) and chlamydia diagnoses per 100,000 population (43% among 15–29-year-olds from 2006 to 2010) [29] within the observation period, we hypothesised that any negative association between the alcopops tax and population-based chlamydia rates might be masked and thought to compare the results with test-based rates. We hypothesised that the association between the tax change and test-based chlamydia rates would occur as an immediate level decrease following the alcopops tax. We postulated the causal mechanism to occur via an overall reduction in young people’s alcohol consumption thereby reducing sexual risk-taking behaviour and the transmission of chlamydia. We proposed the impact model “a priori based on existing literature and knowledge of the intervention and the mechanism by which it is expected to act on the outcome” [35]. Given other alcohol policy evaluation studies on STI rates (e.g., [23]), the abrupt and permanent nature of the intervention [36], evidence for an immediate, significant and sustained decline in RTD consumption unaccounted for by substitution [13,37] and the short window period (2–7 days) between chlamydia exposure and developing viral DNA detectable via polymerase chain reaction [38], the specification of a step function was requisite.

The aim of this study was to investigate the association between the alcopops tax and national chlamydia rates: (i) notification rates (diagnoses per 100,000 population; primary outcome), and (ii) test positivity rates (diagnoses per 100 tests; secondary outcome) among young people using interrupted time series analysis. Gender-specific chlamydia trends in older people were applied as an internal control series. Gender and age-specific consumer price index (CPI)-adjusted income trends were also controlled for as independent variables. Chlamydia was chosen as the outcome variable in this study as it is the most prevalent STI among young Australians [39].

To our knowledge, this is the first alcohol taxation study to have investigated: (a) the association between RTD pricing, a beverage type favoured by young people, and chlamydia rates at a national level, and (b) the use of chlamydia test counts as an alternative denominator to resident population counts, when estimating rates.

## 2. Materials and Methods

### 2.1. Chlamydia Notification Data

Across all Australian states and territories, chlamydia has been a notifiable disease since 1998, and involves mandatory reporting by laboratories (and, in some jurisdictions, doctors) to health departments [31]. Notifications are collated by the National Notifiable Diseases Surveillance System (NNDSS). For this analysis, national monthly notifications of chlamydia, from July 2000 to December 2016 for persons aged 15 years and older were sourced from the NNDSS. Notifications were coded for state/territory of residence, diagnosis month/year, age group (five-year bands) and gender. Monthly trends of notification counts are provided in the Supplementary Materials Figure S1.

**Figure 1:**
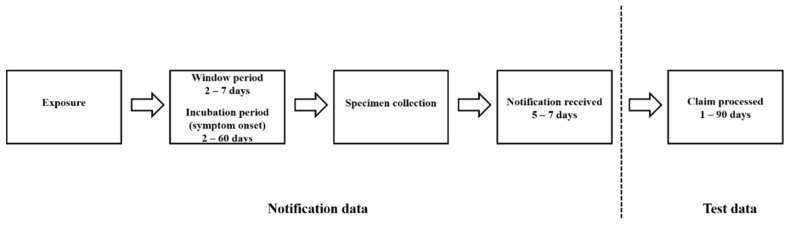
Flowchart from chlamydia exposure to notification (Notification data) and the subsequent financial claim processing of test data (Test data). National Notifiable Diseases Surveillance System defines diagnosis month/year as earliest known of symptom onset date, specimen collection date and notification date. Diagnosis month/year tends to reflect specimen collection or notification date rather than symptom onset date due to the asymptomatic nature of most chlamydia cases.

The NNDSS defines the diagnosis month/year as the earliest known to have occurred among symptom onset date, specimen collection date or notification date. As chlamydia is most often asymptomatic, particularly in females [31], the diagnosis month/year tends to reflect specimen collection or notification date rather than symptom onset date (Figure 1).

### 2.2. Denominator Data: Resident Population and Chlamydia Test Data

Quarterly national estimated resident population by age group and gender from Quarter 3 (Q3) 2000 to Q4 2016 were sourced from the Australian Bureau of Statistics (ABS). Monthly resident population was estimated using linear interpolation within age group and gender. These data were applied as the primary denominator for monthly chlamydia notification rates and trends are provided in the Supplementary Materials Figure S2.

National monthly counts of chlamydia tests were sourced from online publicly available Medicare Benefits Schedule (MBS) Item Statistics Reports for July 2000 to December 2016 by state/territory of residence, test month/year, age group (ten-year bands) and gender. These chlamydia test counts represented tests undertaken in general practice and did not include tests undertaken in public sexual health clinics. In Australia, General Practitioners (GPs) perform the majority of chlamydia tests, as evidenced by the proportion of notifications from this setting. From 2000 to 2010, 75% of chlamydia notifications in NSW (the most populous state/territory) came from general practice settings and this remained stable over time [40]. Notification data were precisely matched to test data age groups (15–24, 25–34, > = 35). Test month/year signified when the financial claim for testing was processed by Medicare, rather than date of the procedure (Figure 1). The following MBS item codes were extracted: 69316, 69317, 69319, 69369, 69370. A 19-month gap in chlamydia-specific test reports occurred from November 2005 to May 2007, due to chlamydia being temporarily grouped into a multiple test MBS item code [41]; linear interpolation within age group and gender was applied to manage data for this period. These data were applied as a secondary denominator to estimate monthly chlamydia test positivity rates and trends are provided in the results.

### 2.3. Outcome Measures: Population-Based and Test Positivity Rates

The primary outcome measure for chlamydia was defined as the national monthly total of gender and age-specific (males 15–24; males 25–34; females 15–24; females 25–34) notifications per 100,000 population in that month. The secondary outcome measure for chlamydia was the monthly total of gender and age-specific notifications per 100 chlamydia tests conducted in that month. The chlamydia series included data from July 2000 to December 2016.

As chlamydia notification data were based on either specimen collection or notification dates and chlamydia test data were based on claim process dates, there was likely to be a lag of at least one month between the datasets (Figure 1). Adjustments were made in order that the timing of notification and test reports matched as closely as possible. There was scant published information on Medicare processing times and there may have been variability over the observation period. In 2013/14, the Department of Human Services (DHS) took an average of 15.5 days to process medical services submitted to Medicare, with 98% processed within three months [42]. An independent audit of DHS in 2017 noted that processing for 70% of pathology services was not automated and required manual intervention, potentially slowing processing times [43]. On this basis, we concluded that notification data best aligned with test data that were processed one month later (e.g., January notifications aligned with February tests and so on).

### 2.4. Socio-Economic Data

Annual per capita total income data by age group and gender (males 18–24; males 25–34; females 18–24; females 25–34) were sourced from the Australian Tax Office (ATO) from financial year (FY) 2000–01 to FY 2016–17. As per capita income data were only available annually from the ATO, monthly data were estimated from July 2000 to December 2016 using linear interpolation within age group and gender. In addition, as per capita income had not been adjusted for inflation, quarterly CPI data from Q3 2000 to Q4 2016 were sourced from the ABS. The monthly CPI was estimated using linear interpolation. With December 2016 set as the reference point, monthly ratios of CPI were calculated. Monthly per capita income by age group and gender was multiplied by these CPI ratios to convert income to real prices to be applied in time series models as independent variables to control for the effect of the GFC on disposable income. These data are provided in the Supplementary Materials Figure S3.

### 2.5. Ready-to-Drink Beverage Consumption Data

Annual per capita consumption of alcohol by beverage type from 2002–03 to 2015–16 was sourced from the ABS [37]. These data were graphed to assist in the interpretation of the results and are provided in the Supplementary Materials Figure S4.

### 2.6. Statistical Analysis

Autoregressive integrated moving average (ARIMA) models were fit to the pre-intervention time-period (94 monthly time points) for the primary and secondary outcome measures. To limit analyst subjectivity, SPSS Statistics’ version 25 expert modeller function, allowing seasonal terms, was used to select best fitting models automatically. The function ensured that model fit was both statistically adequate and the most parsimonious to minimise the likelihood of incorrect inferences [44]. Models of best fit were then applied to their corresponding full time series, while controlling for independent variables. Adequate model fit was confirmed by Stationary R-squared, Ljung-Box Q statistics and inspection of residual autocorrelation and partial autocorrelation function plots.

#### 2.6.1. Independent Variables

Intervention time points were included in all models as dummy independent variables. For chlamydia models, time points from July 2000 to April 2008 before the introduction of the alcopops tax were coded 0 and time points from May 2008 to December 2016 were coded 1. Australian surveys pre- and post-tax reported that RTDs are consumed primarily by young people (aged under 25 years) and considerably less frequently by middle aged and older drinkers [5,6]. In 2007, RTDs were the preferred choice among 14–19-year-old males (37% RTDs, 36% beer, 20% straight spirits, 6% wine) and females (43% RTDs, 33% straight spirits, 18% beer, 6% wine). Preference switched to beer for males (59% beer, 25% wine, 8% straight spirits, 6% RTDs) and wine for females (66% wine, 19% spirits, 13% beer, 9% straight spirits, 9% RTDs) among 40–49-year-olds [6]. Given this, gender-specific chlamydia rates for those aged 35 and older, those much less likely to be affected by the alcopops tax, were included as an internal control. Age- and gender-specific CPI-adjusted per capita income were also included in models as independent variables to control for the effect of the GFC on disposable income.

#### 2.6.2. Lagged Associations

The hypothesised association between the tax change and chlamydia rates may not have been immediately detectable. Lagged associations could occur for many reasons, including the asymptomatic nature of most chlamydia cases [31], and differences between genders in healthcare-seeking behaviour [30,45]. Therefore, lagged associations occurring at three and six months were considered in addition to an immediate association.

#### 2.6.3. Sensitivity Analyses

In order to gauge sensitivity in notification and test month alignment, a two-month realignment was also conducted (e.g., January notifications aligned with March tests and so on). In order to test whether there was a significant level increase in test counts following the alcopops tax that could potentially contribute to a level decrease in test positivity rates, models were fit and applied to gender and age-specific test counts while controlling for gender-specific test counts for those aged 35 and older.

### 2.7. Ethics

This study was conducted in accordance with the National Statement on Ethical Conduct in Human Research and was approved by Human Research Ethics Committees at Curtin University (HR138/2013) and Australian Capital Territory Health (ETHLR.13.070).

## 3. Results

### 3.1. Descriptive Statistics

**Figure 2:**
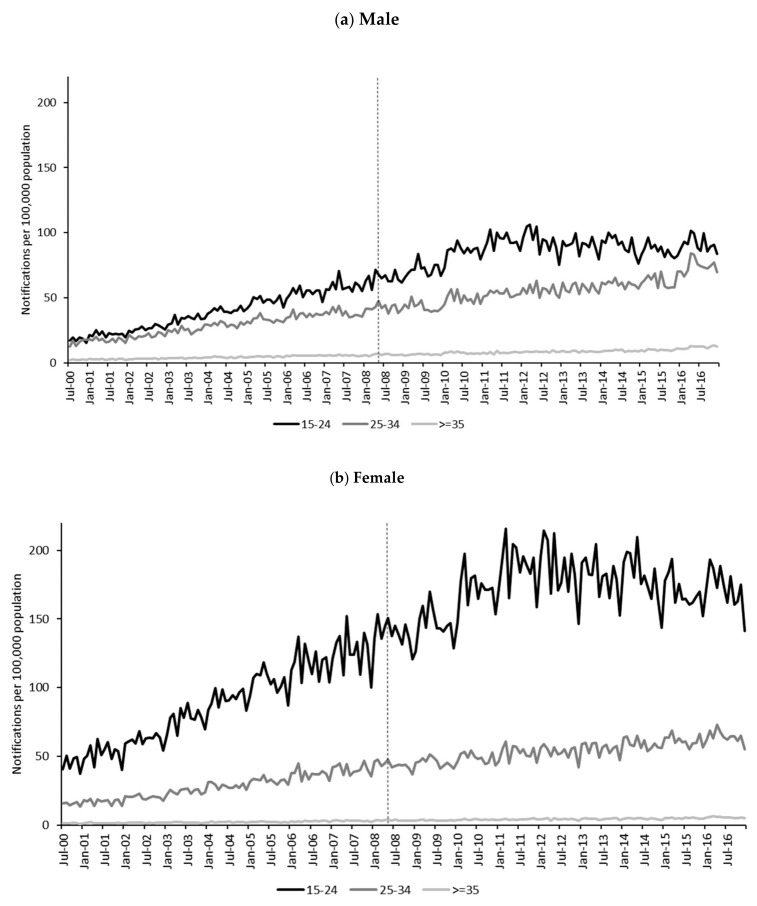
Monthly chlamydia notification rates per 100,000 population (primary outcome) by gender and age group, July 2000 to December 2016. Alcopops tax intervention point indicated by vertical dotted line at May 2008.

**Figure 3:**
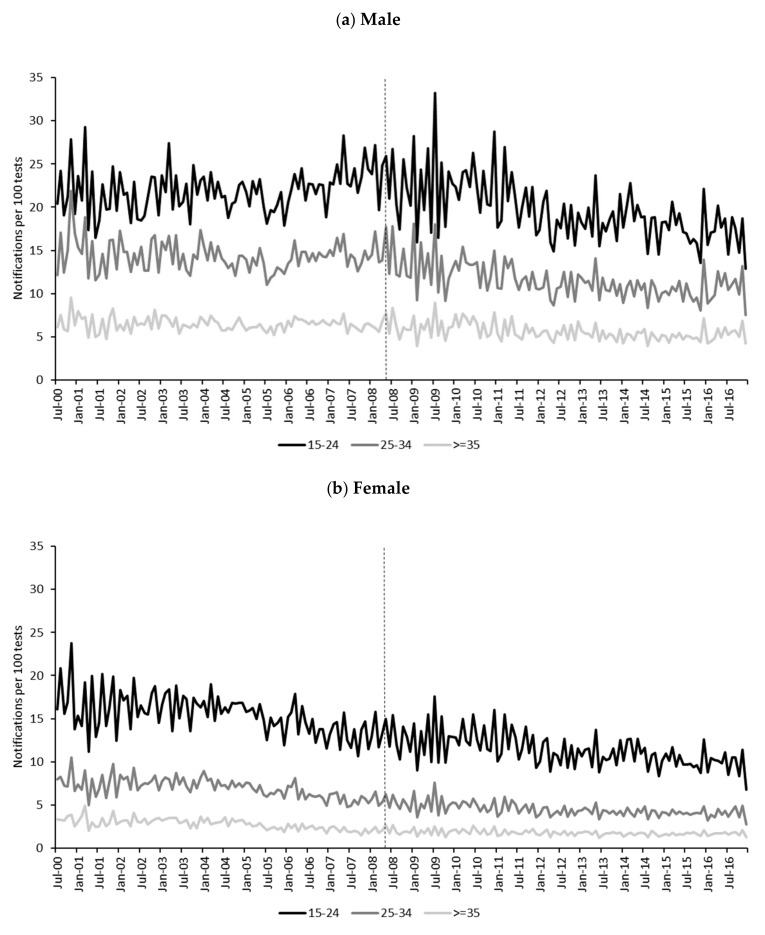
Monthly chlamydia test positivity rates (secondary outcome) by gender and age group, July 2000 to December 2016. Alcopops tax intervention point indicated by vertical dotted line at May 2008. Notification data were aligned with test data that were processed one month later.

Descriptive statistics for monthly chlamydia notification rates per 100,000 population (primary outcome) and test positivity rates (secondary outcome) by age group and gender are presented in Table 1. Monthly notification rate trends per 100,000 population (primary outcome) and test positivity rates (secondary outcome) by age group and gender are presented in Figure 2 and Figure 3. Trends in chlamydia rates varied across gender and age groups, and between the primary and secondary outcome measures.

#### 3.1.1. Primary Outcome Measure

From July 2000 to December 2016, there were 960,694 chlamydia notifications (Table 1). Of these, 555,782 (58%) were among females and 848,844 (88%) were among those under 35 years old. Median monthly notification rates per 100,000 population were highest among 15–24-year-old females pre- (90 per 100,000) and post- (173 per 100,000) alcopops tax. Monthly notification rate trends per 100,000 population (Figure 2) were highest among the 15–24 age group and lowest among the 35 and older age group. Among 15–24-year-olds, the population-based rates were markedly and consistently higher for females compared to males, but trends were similar with a steady increase until 2011, at which point they levelled off. For those aged 25–34 years and 35 and older there were steady increases in population-based notification rates without any obvious decline or levelling off apparent from visual inspection.

#### 3.1.2. Secondary Outcome Measure

Median monthly chlamydia test positivity rates (Table 1) were highest among 15–24-year-old males pre- (22 per 100 tests) and post- (19 per 100) alcopops tax. Monthly test positivity rates (Figure 3) demonstrated markedly different trends and reversed gender ratios compared to rates generated per 100,000 population. Similar to the primary outcome measure, test positivity rates were highest among the 15–24 age group and lowest among those 35 and older; however, rates were consistently higher among males than females for all age groups. Among males aged 15–24 and 25–34 years old, trends appeared relatively stable until around 2008, at which point visual inspection suggested a declining trend until 2016. Among males 35 and older, trends were relatively stable over the observation period. There were relatively steady decreases in test positivity rates among females of all ages over the observation period.

**Figure 4:**
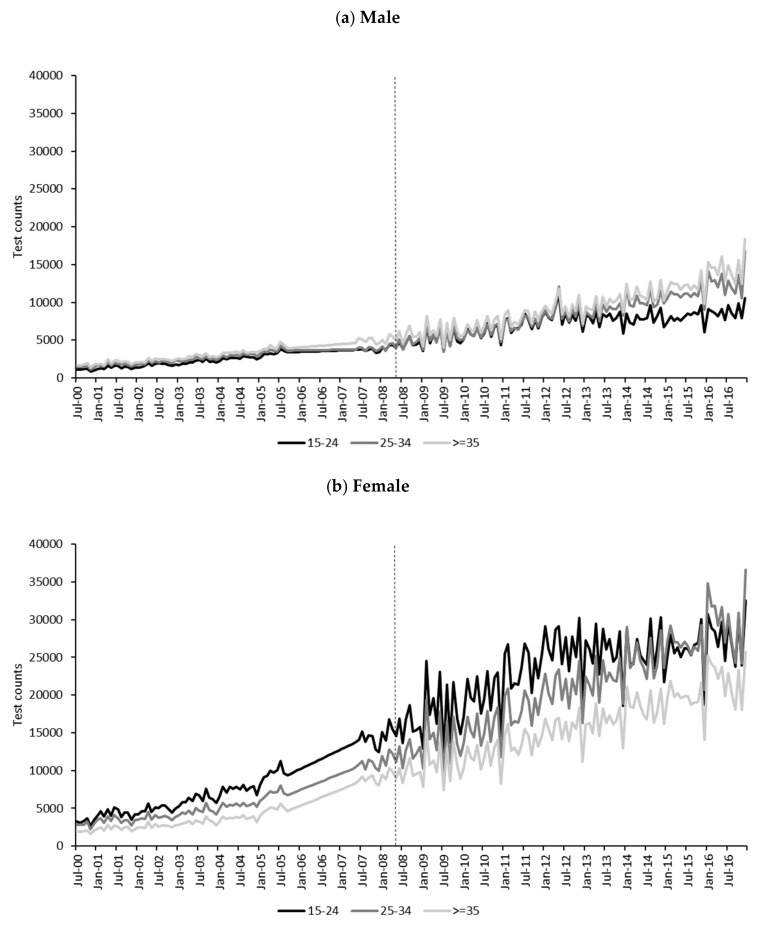
Monthly chlamydia test counts by gender and age group, July 2000 to December 2016. Alcopops tax intervention point indicated by vertical dotted line at May 2008. Test counts interpolated within age group and gender between November 2005 and May 2007 due to missing data. Test counts shifted back by 1 month to represent date of service better (rather than date processed).

Trends in monthly chlamydia test counts (Figure 4) were markedly and consistently higher for females compared to males for all age groups. Among males, test counts were highest for those 35 and older and lowest for 15–24-year-olds. Trends in test counts for males of all ages increased steadily until 2008, at which point there appeared to be an increase in slope. Males aged 15–24 years old then levelled off around 2012. Among females aged 25–34 years and 35 and older, there were steady increases in test counts over the observation period. For females aged 15–24 years old, test count trends had a steady increase until around 2012, at which point they levelled off. Supplementary Materials include monthly trends of chlamydia notification counts and estimated resident population (Figures S1 and S2), CPI-adjusted per capita total income (Figure S3) and annual per capita consumption of alcohol by beverage type (Figure S4). Trends in annual per capita consumption of RTDs show a marked reduction in consumption between 2007–08 and 2008–09, followed by a steady decline through to 2016.

### 3.2. ARIMA Models

#### 3.2.1. Primary Outcome Measure

ARIMA model results for the primary outcome, chlamydia notification rates per 100,000, are shown in Table 2. All models demonstrated an adequate fit. None of the gender and age-specific population-based rates indicated a significant immediate or lagged association with the alcopops tax.

#### 3.2.2. Secondary Outcome Measure

ARIMA model results for the association between the alcopops tax and monthly chlamydia test positivity rates are shown in Table 3. All models demonstrated an adequate fit. We found an immediate decrease in test positivity rates for 25–34-year-old males (−0.726, SE = 0.311, *p* = 0.02; 27% reduction) that remained detectable up to a lag of six months—on average, 112 fewer chlamydia notifications per month following the alcopops tax, from May 2008 to December 2016. Among 15–24-year-old males, a decrease in test positivity rates was detected at a six month lag (−1.439, SE = 0.688, *p* = 0.04; 31% reduction)—on average, 182 fewer notifications per month from November 2008 to December 2016. There were no significant immediate or lagged associations with the alcopops tax for females in either age group.

#### 3.2.3. Sensitivity Analyses

Results from sensitivity analyses based on a two-month realignment of the test to the notification month supported our findings for the secondary outcome and are presented in the Supplementary Materials Table S1. The parameter estimates for 15–24-year-old males almost reached significance for an immediate association (−1.221, SE = 0.618, *p* = 0.05). Results from sensitivity analyses on the association between the alcopops tax and test counts were not significant for any gender or age combination. They supported our findings for the secondary outcome and are presented in the Supplementary Materials Table S2.

**Table 2:** Autoregressive integrated moving average (ARIMA) model results of the association between introduction of the alcopops tax and monthly chlamydia notification rates per 100,000 population (primary outcome) by age group and gender, July 2000 to December 2016.

							Immediate			3 Month Lag			6 Month Lag		
Age	Gender	Model	SR^2^	Q	df	*p*	Est	SE	*p*	Est	SE	*p*	Est	SE	*p*
15–24	Male	(0,1,1) (0,1,1)_12_	0.61	13.65	16	0.63	0.004	0.003	0.22	0.003	0.003	0.35	0.002	0.003	0.61
	Female	(0,1,1) (0,1,0)_12_	0.45	19.99	17	0.28	-0.002	0.005	0.71	−0.002	0.005	0.73	−0.001	0.005	0.84
25–34	Male	(0,1,1) (0,1,0)_12_	0.45	19.24	17	0.32	-0.002	0.004	0.60	−0.004	0.005	0.41	−0.004	0.005	0.34
	Female	(0,0,0) (0,1,0)_12_	0.25	27.73	18	0.07	-0.032	0.027	0.23	−0.036	0.027	0.18	−0.021	0.027	0.44

*p < 0.05. All ARIMA models controlled for gender-specific chlamydia rates for the 35 and older age group and age- and gender-specific total income. All time series were log-transformed before modelling. Stationary R^2^ for immediate effect models. Ljung-Box test (Q) based on first 18 autocorrelation lags of the pre-alcopops tax model residuals.

**Table 3:** ARIMA model results of the association between introduction of the alcopops tax and monthly chlamydia test positivity rates (secondary outcome) by age group and gender, July 2000 to December 2016.

							Immediate			3 Month Lag			6 Month Lag		
Age	Gender	Model	SR^2^	Q	df	*p*	Est	SE	*p*	Est	SE	*p*	Est	SE	*p*
15–24	Male	(0,0,2) (1,0,0)_12_	0.69	21.19	16	0.17	−0.865	0.688	0.21	−1.181	0.689	0.09	−1.439*	0.688	0.04
	Female	(0,0,0) (1,0,0)_12_	0.80	33.91*	17	0.01	−0.307	0.436	0.48	−0.199	0.448	0.66	−0.196	0.456	0.67
25–34	Male	(0,0,0) (1,0,0)_12_	0.80	16.67	17	0.48	−0.726*	0.311	0.02	−0.970*	0.306	<0.01	−1.168*	0.304	<0.001
	Female	(1,0,1) (1,0,0)_12_	0.90	18.08	15	0.26	−0.192	0.161	0.23	−0.136	0.161	0.40	−0.197	0.160	0.22

*p < 0.05. ARIMA models controlled for gender-specific chlamydia rates for the 35 and older age group and age- and gender-specific total income. Notification data were aligned with test data that were processed one month later. Stationary R^2^ for immediate effect models. Ljung-Box test (Q) based on first 18 autocorrelation lags of the pre-alcopops tax model residuals.

## 4. Discussion

We were unable to detect an association between the alcopops tax and our primary outcome measure of chlamydia notification rates based on population denominators. However, chlamydia rates estimated on the basis of the number of tests conducted were markedly different to the population-based trends and we found a significant association, in the expected direction, between the alcopops tax and test positivity rates among males aged between 15 and 24 (31% reduction, six month lag) and between 25 and 34 (27% reduction, immediate) years old. The results remained largely unchanged in the sensitivity analyses. We estimate a total of 16,615 chlamydia cases were prevented over eight years among 15–24-year-old males and 11,891 cases were prevented over eight and a half years among 25–34-year-old males following the alcopops tax and its impact on alcopop prices. These estimates of cases that were prevented are conservative, as most chlamydia infections are asymptomatic and not diagnosed. Among 15–29-year-olds in Australia, it is estimated that for every notification, there are 3.5 cases that go undiagnosed [31]. If untreated, chlamydia potentially leads to reproductive and neonatal morbidity, such as pelvic inflammatory disease, infertility, ectopic pregnancy, preterm labour, low birth weight and perinatal mortality [46,47]. Current national primary care guidelines suggest annual testing of all sexually active people under 30 years old [48]. More frequent testing is recommended for higher-risk gay and bisexual men and sex workers [48].

What is clear from the results is that taking the frequency of tests into account when estimating chlamydia rates substantially altered the trends compared to rates based on resident population. Test positivity rates were consistently higher among males than females for all age groups because fewer chlamydia tests were conducted in males than females and, thus, tests in males were more likely to be positive. Test counts increased considerably over the observation period, reflecting greater awareness and screening programs in general practice [34,49]. Models built on notifications per 100,000 population did not detect any association between the tax and chlamydia rates. Sensitivity analyses on the association between the alcopops tax and test counts were not significant for any gender or age combination, supporting our findings for the secondary outcome. This suggests that the test data may be a better proxy of the affected population than resident population and test positivity rates may be a more sensitive and appropriate means for evaluating alcohol policy effects than population-based notification rates, which do not take into account test patterns and differences in test behaviour across the population [29,30].

Our finding that the alcopops tax significantly reduced chlamydia test positivity rates among males aged 15–24 (lagged) and 25-34 (immediate) adds to the findings from studies from New South Wales [8], Western Australia and Victoria [16], all of which showed reductions in injury rates among males in these age groups following the introduction of the tax. It is of interest that we did not find an association between the alcopops tax and chlamydia test positivity rates among females, as the NSW study found that both the GST and alcopops tax had strong negative associations with ED attendances in 18–24-year-old females [8]. The Queensland studies found no association between the alcopops tax and ED attendance or hospitalisation among 15–29-year-olds for either gender [17,18,19].

In the years between the GST and the alcopops tax (when prices declined), although a greater proportion of females among 14–19-year-olds drank at risky levels on single occasions (>50g) than males (>70g), a greater proportion of males among 20–29 and 30–39-year-olds drank at risky levels on single occasions than females [6]. According to industry sources, RTDs based on dark spirits and typically sold in cans (e.g., bourbon whiskey and cola) made up three quarters of the alcopop market [50]. Young and underage males (14–19 years old) who engage in single occasion risky drinking (>70g) have a strong preference for these dark spirit-based RTDs (74%) [51]. Young and underaged females also have a preference for RTDs when drinking at risky levels (> 50g) but they generally choose white spirits that are typically sold in bottles (78%). Males in their twenties tend to move away from RTDs and more towards regular strength beer and straight spirits; nevertheless, consumption of dark spirit-based RTDs remains prevalent, with 58% of 20–24 and 44% of 25–29-year-old males preferring them when engaging in risky drinking occasions. Female RTD consumption also declines with age but is still reported as a preferred beverage on risky drinking occasions by 63% of 20–24 and 42% of 25–29-year-olds [51]. Although the overall trends in chlamydia test positivity rates appeared similar for the two age groups in both genders—stable and then declining from 2008 for males and an overall steady decrease for females—there was notably larger monthly variability during the 18 months immediately after the tax, which may have reduced statistical power. It is also likely that the 15–24 year age group in both genders is more heterogenous in its drinking and sexual behaviours compared to the 25–34 year age group, as it includes teenagers and likely a proportion of high risk underage drinkers (<18 years old) who may take longer to respond to policy change. The test data applied in our study were only available in pre-set age groups, thereby limiting our ability to separate teenagers and those in their early twenties (71% of 15–24 male notifications were among 20–24-year-olds) and those in their late twenties and early thirties (66% of 25–34 male notifications were among 25–29-year-olds) for analysis. Lagged associations on chlamydia test positivity rates among 15–24 and 25–34-year-old males following the alcopops tax may have been related to the asymptomatic nature of most chlamydia cases [31], which can delay testing and detection. Males are also less likely than females to present to a GP with a sexual health complaint, although more likely to be symptomatic when they do attend, and less likely than females to follow through with an STI test should a GP request one [30,45].

In order to accurately inform future decision making, alcohol policies and their effects on public health must be well evaluated using robust study designs and sensitive measures. When randomised controlled trials are not feasible, interrupted time series analysis offers a strong alternative study design. Using a novel approach to constructing STI rates in an alcohol taxation study, we found evidence of an association between the alcopops tax and chlamydia test positivity rates among 15–24 and 25–34-year-old males; this association would have remained undetected had we relied only on standard methods. Other strengths of this study were its national focus, use of high quality mandatory notification data, application of an internal control series (males and females > = 35 years), adjustment for gender and age-specific CPI-adjusted per capita income to control for the effect of the GFC on disposable income, and sensitivity analyses.

Despite the strong study design, we note a number of limitations. This is an ecological study and, as such, it may not reflect the behaviour of individuals. The publicly available chlamydia test data represented tests undertaken in general practice settings and did not include tests undertaken at public sexual health clinics [31]. However, the majority of tests are conducted in general practice, as evidenced by the higher proportion of notifications from this setting [40]. Assumptions were necessary to align the notification and test data as closely as possible in the calculation of chlamydia test positivity rates, and having the test service date rather than the claim process date would have allowed for more accurate alignment. Missing data from the chlamydia test series required interpolation and data were only available in ten-year age groups thereby limiting our ability to separate out teenage/underage drinkers. Longer national chlamydia notification time series would have facilitated evaluation of the GST in addition to the alcopops tax (e.g., [8])– if significant increases in chlamydia test positivity rates were found among males following the GST it would have strengthened our findings. We also considered gonorrhoea as a potential outcome measure, as national data collection has been mandated for longer than chlamydia, but it was deemed not suitable for this evaluation as it is mainly concentrated in gay and bisexual men, and young Aboriginal people living in remote areas of Australia [31]. A geographic control would have strengthened the analysis; however, the intervention was introduced nationwide. There may have been other confounding factors, such as community-level sexual health promotion campaigns that may have increased safer sex practices, that we were unable to control for. Unfortunately, sexual risk behaviour data were not available by gender and age group around the time of the alcopops tax, however, there are reports of small but significant increases in condom use by heterosexual men in their most recent sexual encounter between national surveys taken in 2001–02 (25%) and 2012–13 (29%) [52].

## 5. Conclusions

The 2008 alcopops tax was an attempt to remedy a regulatory loophole created by the introduction of the GST in 2000 and to reduce sales of ready-to-drink beverages to vulnerable young people. This study adds to the evidence base supporting the use of alcohol taxation to reduce health-related harms experienced by young people and offers a novel method in calculating sexually transmitted infection rates for policy evaluation.

## Figures and Tables

**Table 1:** Descriptive statistics for monthly chlamydia rates per 100,000 (primary outcome) and per 100 tests (secondary outcome) pre- and post-alcopops tax intervention (27th April 2008), by age group and gender.

Age	Gender	Notifications		Pre-Alcopops Tax		Post-Alcopops Tax	
				Jul 2000 to April 2008 *(N = 94)*		May 2008 to Dec 2016 *(N = 104)*	
		*n*	*%*	Median	IQR	Median	IQR
**Per 100,000 population**							
15–24	Male	195,986	20.4	39.6	26.0–53.0	88.0	81.5–93.4
	Female	385,047	40.1	89.5	62.4–110.1	173	159–187
25–34	Male	135,960	14.2	29.3	20.5–35.5	56.0	49.2–61.4
	Female	131,851	13.7	27.8	20.3–36.3	55.3	47.9–59.7
35 and older	Male	72,966	7.6	4.0	3.1–5.3	8.4	7.3–9.4
	Female	38,884	4.1	2.1	1.6–2.6	4.3	3.7–4.9
Total		960,694	100	18.3	12.6–23.2	36.3	33.1–38.4
**Per 100 tests**							
15–24	Male	195,986	20.4	21.8	20.1–23.5	18.9	17.3–22.1
	Female	385,047	40.1	15.4	13.8–16.9	11.0	10.0–12.6
25–34	Male	135,960	14.2	14.2	13.0–15.3	11.3	10.2–12.6
	Female	131,851	13.7	6.9	6.1–7.8	4.3	4.0–4.9
35 and older	Male	72,966	7.6	6.4	6.0–7.0	5.4	4.9–6.2
	Female	38,884	4.1	2.8	2.3–3.2	1.7	1.6–2.0
Total		960,694	100	10.9	10.0–11.8	7.8	7.0–9.0

*n* = number of notifications; *N* = number of time points; IQR = interquartile range. Notification data were aligned with test data that were processed one month later.

## Supplementary Materials

The following are available online at https://www.mdpi.com/1660-4601/17/4/1343/s1. Figure S1: Monthly chlamydia notification counts by gender and age group, July 2000 to December 2016. Alcopops tax intervention point indicated by dotted line at May 2008, Figure S2: Monthly estimated resident population by gender and age group, July 2000 to December 2016. Alcopops tax intervention point indicated by dotted line at May 2008. Quarterly estimated resident population was interpolated within age group and gender to achieve monthly data points, Figure S3: Monthly CPI-adjusted per capita total income ($) by age group and gender, July 2000 to Dec 2016. Alcopops tax intervention point indicated by dotted line at May 2008, Figure S4: Annual litres of pure alcohol consumed per capita by beverage type, 2002-03 to 2015-16. Alcopops tax intervention point indicated by dotted line at 2007-08, Table S1: ARIMA model results of the association between introduction of the alcopops tax and monthly chlamydia test positivity rates (secondary outcome) by age group and gender with a two-month realignment of test to notification month, July 2000 to December 2016, Table S2. ARIMA model results of the association between introduction of the alcopops tax and monthly chlamydia test counts by age group and gender, July 2000 to December 2016.
