# Supplementary material for: The Association between the Australian Alcopops Tax and National Chlamydia Rates among Young People—an Interrupted Time Series Analysis
Source: Int J Environ Res Public Health. 2020 Feb 19;17(4):1343. doi: 10.3390/ijerph17041343 (PMC7068511; doi:10.3390/ijerph17041343)
Supplement: Supplementary file 1 [file ijerph-17-01343-s001.pdf]

International Journal of
Environmental Research and Public Health

MDPI

Supplementary Materials

a) Male

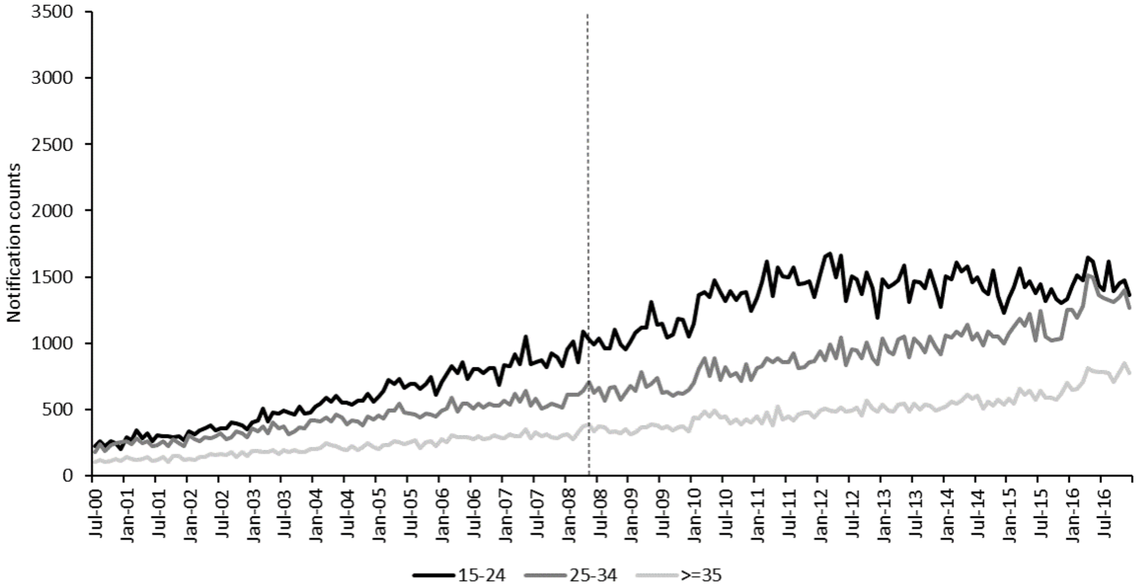

b) Female

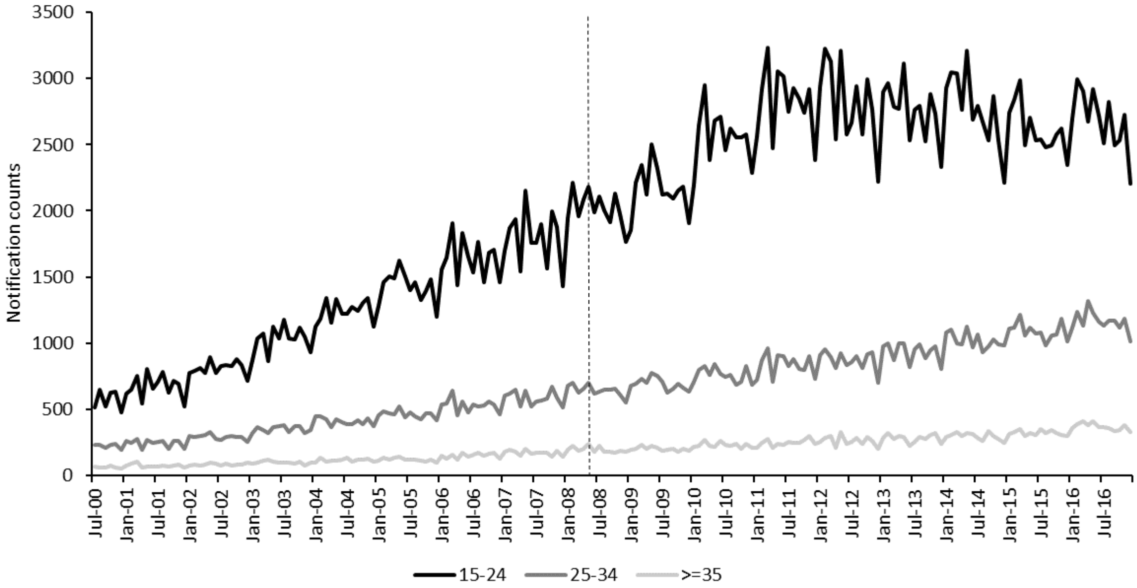

Figure S1. Monthly chlamydia notification counts by gender and age group, July 2000 to December 2016. Alcopops tax intervention point indicated by vertical dotted line at May 2008.

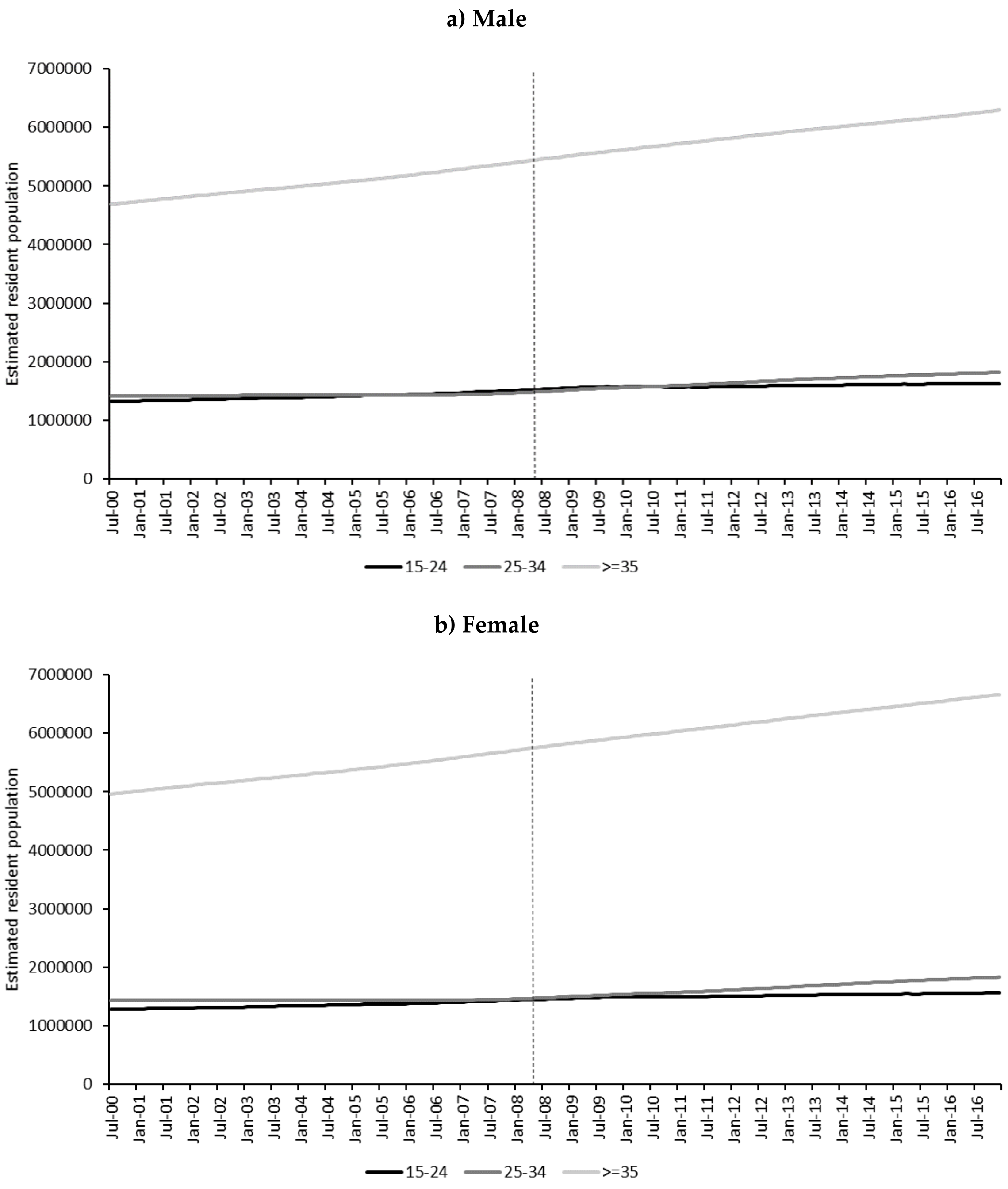

Figure S2. Monthly estimated resident population by gender and age group, July 2000 to December 2016. Alcopops tax intervention point indicated by vertical dotted line at May 2008. Quarterly estimated resident population was interpolated within age group and gender to achieve monthly data points.

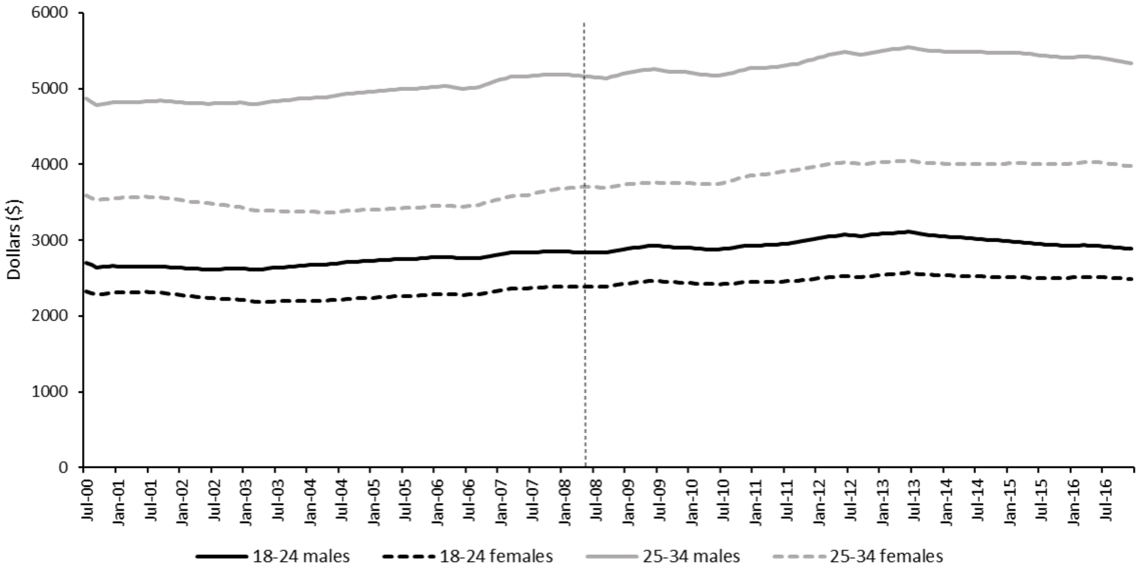

Figure S3. Monthly CPI-adjusted per capita total income ($) by age group and gender, July 2000 to Dec 2016. Alcopops tax intervention point indicated by vertical dotted line at May 2008.

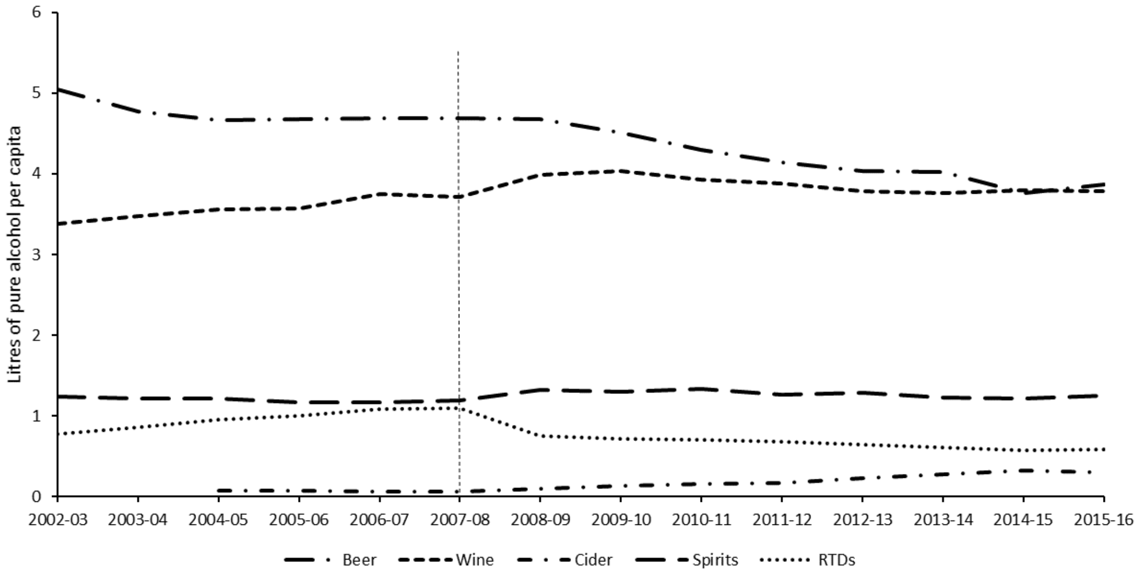

Figure S4. Annual litres of pure alcohol consumed per capita by beverage type, 2002-03 to 2015-16. Alcopops tax intervention point indicated by vertical dotted line at 2007-08.

Table S1. ARIMA model results of the association between introduction of the alcopops tax and monthly chlamydia test positivity rates (secondary outcome) by age group and gender with a two-month re-alignment of test to notification month, July 2000 to December 2016.

							**Immediate**			**3 Month Lag**			**6 Month Lag**		
Age	Gender	Model	SR²	Q	df	*p*	Est	SE	*p*	Est	SE	*p*	Est	SE	*p*
15–24	Male	(0,0,0) $(1,0,0)_{12}$	0.64	33.40*	17	0.01	-1.221	0.618	0.05	-1.159	0.617	0.06	-1.311*	0.614	0.03
	Female	(3,0,0) $(1,0,0)_{12}$	0.80	22.64	16	0.12	-0.300	0.447	0.50	-0.145	0.458	0.75	-0.147	0.467	0.75
25–34	Male	(0,0,3)	0.79	22.49	17	0.17	-0.796*	0.313	0.01	-1.017*	0.305	<0.01	-1.157*	0.301	<0.001
	Female	(3,0,1) $(1,0,0)_{12}$	0.89	12.78	14	0.54	-0.199	0.180	0.27	-0.118	0.180	0.51	-0.204	0.180	0.26

*p<0.05. ARIMA models controlled for gender-specific chlamydia rates for the 35 and older age group and age- and gender-specific total income. Notification data were aligned with test data that were processed two months later. Stationary R^2 for immediate effect models. Ljung-Box test (Q) based on first 18 autocorrelation lags of the pre alcopops tax model residuals.

Table S2. ARIMA model results of the association between introduction of the alcopops tax and monthly chlamydia test counts by age group and gender, July 2000 to December 2016.

							**Immediate**			**3 Month Lag**			**6 Month Lag**		
Age	Gender	Model	SR²	Q	df	*p*	Est	SE	*p*	Est	SE	*p*	Est	SE	*p*
15–24	Male	(0,1,1) $(1,0,0)_{12}$	0.54	21.16	16	0.17	0.003	0.005	0.55	0.002	0.006	0.77	0.000	0.006	0.97
	Female	(0,1,1) $(1,0,1)_{12}$	0.60	21.92	15	0.11	0.002	0.003	0.49	0.002	0.003	0.56	0.001	0.003	0.72
25–34	Male	(0,1,1) $(1,0,0)_{12}$	0.56	13.64	16	0.63	0.005	0.004	0.17	0.005	0.004	0.19	0.005	0.004	0.27
	Female	(0,1,1) $(0,1,1)_{12}$	0.72	19.41	16	0.25	-0.002	0.002	0.29	-0.001	0.002	0.46	-0.001	0.001	0.58

*p<0.05. ARIMA models controlled for gender-specific test counts for the 35 and older age group. Natural logarithm applied to age- and gender-specific test counts. Test counts shifted back by 1 month to represent date of service better (rather than date processed). Stationary R^2 for immediate effect models. Ljung-Box test (Q) based on first 18 autocorrelation lags of the pre alcopops tax model residuals.
